# Dietary Arginine Regulates Severity of Experimental Colitis and Affects the Colonic Microbiome

**DOI:** 10.3389/fcimb.2019.00066

**Published:** 2019-03-26

**Authors:** Kshipra Singh, Alain P. Gobert, Lori A. Coburn, Daniel P. Barry, Margaret Allaman, Mohammad Asim, Paula B. Luis, Claus Schneider, Ginger L. Milne, Helen H. Boone, Meghan H. Shilts, M. Kay Washington, Suman R. Das, M. Blanca Piazuelo, Keith T. Wilson

**Affiliations:** ^1^Division of Gastroenterology, Hepatology, and Nutrition, Department of Medicine, Vanderbilt University Medical Center, Nashville, TN, United States; ^2^Center for Mucosal Inflammation and Cancer, Vanderbilt University Medical Center, Nashville, TN, United States; ^3^Veterans Affairs Tennessee Valley Healthcare System, Nashville, TN, United States; ^4^Department of Pharmacology, Vanderbilt University Medical School, Nashville, TN, United States; ^5^Division of Clinical Pharmacology, Vanderbilt University Medical School, Nashville, TN, United States; ^6^Division of Infectious Diseases, Vanderbilt University Medical Center, Nashville, TN, United States; ^7^Department of Pathology, Microbiology, and Immunology, Vanderbilt University Medical Center, Nashville, TN, United States

**Keywords:** arginine, experimental colitis, gut microbiota, alternative therapies, inflammatory bowel diseases

## Abstract

There is great interest in safe and effective alternative therapies that could benefit patients with inflammatory bowel diseases (IBD). L-arginine (Arg) is a semi-essential amino acid with a variety of physiological effects. In this context, our aim was to investigate the role of dietary Arg in experimental colitis. We used two models of colitis in C57BL/6 mice, the dextran sulfate sodium (DSS) model of injury and repair, and *Citrobacter rodentium* infection. Animals were given diets containing (1) no Arg (Arg^0^), 6.4 g/kg (Arg^NL^), or 24.6 g/kg Arg (Arg^HIGH^); or (2) the amino acids downstream of Arg: 28 g/kg L-ornithine (Orn^HIGH^) or 72 g/kg L-proline (Pro^HIGH^). Mice with DSS colitis receiving the Arg^HIGH^ diet had increased levels of Arg, Orn, and Pro in the colon and improved body weight loss, colon length shortening, and histological injury compared to Arg^NL^ and Arg^0^ diets. Histology was improved in the Arg^NL^ vs. Arg^0^ group. Orn^HIGH^ or Pro^HIGH^ diets did not provide protection. Reduction in colitis with Arg^HIGH^ diet also occurred in *C. rodentium*-infected mice. Diversity of the intestinal microbiota was significantly enhanced in mice on the Arg^HIGH^ diet compared to the Arg^NL^ or Arg^0^ diets, with increased abundance of Bacteroidetes and decreased Verrucomicrobia. In conclusion, dietary supplementation of Arg is protective in colitis models. This may occur by restoring overall microbial diversity and Bacteroidetes prevalence. Our data provide a rationale for Arg as an adjunctive therapy in IBD.

## Introduction

Inflammatory bowel disease (IBD), which includes ulcerative colitis (UC) and Crohn's disease (CD), remains a major public health problem (Torres et al., 2017; Ungaro et al., 2017). There are nearly 2 million people in the USA afflicted with IBD, and its prevalence continues to increase in the USA (Shivashankar et al., 2017) and dramatically worldwide, especially in India and China (Singh et al., 2017). Both UC and CD severely compromise the quality of life of affected individuals and IBD represents a major risk for development of cancer since 20% of these patients can develop colitis-associated carcinogenesis (Bernstein et al., 2001; Terzic et al., 2010).

The etiology of IBD is undoubtedly multifactorial and is thought to result from the complex interplay between genetic susceptibility (Liu and Stappenbeck, 2016), the gut microbiota (Frank et al., 2007), and environmental factors (Kaser et al., 2010), resulting in a dysregulated mucosal immune response. In this context, biologic therapies for IBD, which include anti-TNFs (Sandborn et al., 2012), anti-IL-12/23p40 (Mannon et al., 2004), anti-integrins (Engel et al., 2018), and others have been deployed to limit the chronic colonic inflammation. However, these agents induce remission in only half of patients, are very expensive, and may have many side effects. Alternative therapies that would be safe, well-tolerated, cost-effective, and rationally-based that could beneficially impact IBD patients would be ideal.

Epithelial and myeloid cells in the inflamed intestinal mucosa play an important role in the inception, chronicity, and severity of IBD (Torres et al., 2017; Ungaro et al., 2017). These cells develop a non-specific innate immune response to microbiota components that favor acute inflammation and stimulate the initiation of an adaptive immune activation. Inducible enzymes involved in the transport and metabolism of L-arginine (Arg) are altered in patients with IBD and in murine models of colonic inflammation, and play a critical role in the regulation of the inflammatory processes. We have found that expression of the Arg transporter, solute carrier family 7 member 2 (SLC7A2, also known as cationic amino acid transporter 2) is reduced in the colonic mucosa of patients with active UC or CD, and that colon tissue Arg levels are inversely correlated with disease severity (Coburn et al., 2016). Moreover, we have reported that mice lacking the gene *Slc7a2* exhibit more severe dextran sulfate sodium (DSS)-induced colonic injury and innate and Th17 response than wild-type animals (Singh et al., 2013), and also develop increased colitis-associated carcinogenesis (Coburn et al., 2018), suggesting that Arg uptake is important to dampen inflammation and carcinogenesis.

L-Arg is a substrate for four enzymes, namely nitric oxide (NO) synthase (NOS), arginase, glycine amidinotransferase (GATM), and arginine decarboxylase (Morris, 2004). Although clinical and experimental investigations have shown that the inducible isoform of NOS, NOS2, is expressed in the inflamed mucosa in IBD patients (Rachmilewitz et al., 1995) or in animals with experimental colitis (Hokari et al., 2001), the role of NO in colitis remains a subject of controversy. Studies have shown that the level of NO is correlated with disease severity (Krieglstein et al., 2001), whereas other have demonstrated that NO has protective effects in colitis (Yoshida et al., 2000). Arginase and GATM are essential enzymes that exhibit important roles in colitis (Gobert et al., 2004; Turer et al., 2017). Arginase metabolizes Arg into urea and L-ornithine (Orn) whereas GATM converts Arg into creatine and Orn (Morris, 2004). Orn is converted into the polyamine putrescine by ornithine decarboxylase (ODC); then the two other polyamines, spermidine, and spermine, are sequentially generated from putrescine (Pegg, 2016). Additionally, the enzyme arginine decarboxylase cleaves Arg into CO_2_ and agmatine, which is then converted to putrescine by agmatinase (Morris, 2004). In mice infected with the intestinal pathogen *C. rodentium*, we have found that the arginase/ODC metabolic pathway protects mice from colitis (Gobert et al., 2004; Hardbower et al., 2017). Furthermore, Orn can also be converted by ornithine aminotransferase into L-proline (Pro), which is a precursor in collagen synthesis and thus supports wound healing (Singh et al., 2012). All together, these data suggest that the enhancement of Arg metabolism may protect from colitis.

The most common dietary sources of Arg are meat, fish, dairy products, and nuts (Visek, 1986; Hu et al., 1998) and Arg supplementation is generally considered to be safe (Collier et al., 2005; Shao and Hathcock, 2008). Because it acts as a vasodilator through NO synthesis, Arg is used as a complementary medicine to help in the treatment of hypertension (Dong et al., 2011). Clinical investigations have also shown that Arg ameliorates glucose metabolism and insulin sensitivity in type 2 diabetes (Lucotti et al., 2006). Hence, in the current report, we analyzed the effect of specific diets containing various amount of Arg in two models of experimental colitis. We show that depletion of L-Arg is detrimental in both models, whereas an Arg-rich diet protects animals from injury and inflammation. Surprisingly, the beneficial effect of Arg is not dependent on the neosynthesis of Orn or Pro. Rather, we found that Arg supplementation restores the diversity of the intestinal microbiota.

## Materials and Methods

### Diets and Experimental Models of Colitis

The regular 5L0D chow was obtained from LabDiet. The customized amino-acid-defined AIN-76A diet, in which the protein casein was replaced with equivalent amounts of purified amino acids, was purchased from Bio-Serv. These different diets are described in Table 1.

**Table 1:** Amino acid concentration in the diets used in the study.

**Diet**	**Arg (g/kg)**	**Orn (g/kg)**	**Pro (g/kg)**	**Calories (kcal/g)**
5L0D	6.4	ND	18	4.09
AIN-76A Arg^0^	0	ND	18	3.77
AIN-76A Arg^NL^	6.4	ND	18	3.79
AIN-76A Arg^HIGH^	24.6	ND	18	3.86
AIN-76A Orn^NL^	6.4	ND	18	3.79
AIN-76A Orn^HIGH^	6.4	28	18	3.90
AIN-76A Pro^NL^	6.4	ND	18	3.79
AIN-76A Pro^HIGH^	6.4	ND	72	4.01

*ND, not detectable*.

All the animals were bred in our animal facility. Age-matched C57BL/6 male mice (6–7 weeks old) maintained on the 5L0D diet were given the AIN-76A Arg^NL^ diet for 7 days prior to the induction of two models of colitis (Singh et al., 2016; Gobert et al., 2018): (i) Animals were treated or not with 2.5% DSS (mol. wt. 36,000–50,000; TdB Consultancy) in the drinking water for 5 days; DSS was then removed and mice were kept for 5 more days on regular drinking water and on AIN-76A diet containing different concentrations of Arg, Pro, or Orn. (ii) Mice fed the AIN-76A diet containing different concentrations of Arg were infected by oral gavage with 0.1 ml of LB broth containing 5 × 10^8^ *C. rodentium* DBS100 (Barthold et al., 1976) under exponential growth phase or with broth, and euthanized after 14 days after being maintained on each of the three different Arg diets.

In both models, mice were weighed and monitored daily, and those that showed extreme distress, became moribund, or lost more than 20% of initial body weight were euthanized. Feces were collected during the time course of colitis. After sacrifice, colons were removed, measured, cut longitudinally, cleaned, weighed, and Swiss-rolled for histology. Three proximal and distal 2 mm pieces were used for amino acid analysis, polyamine analysis, or determination of *C. rodentium* colonization by culturing serial dilution of ground tissues on Luria-Bertani agar plates (Singh et al., 2016; Gobert et al., 2018).

### Assessment of Histological Injury

Swiss-rolled colons were fixed in formalin and embedded in paraffin, and 5 μm sections were stained with hematoxylin and eosin (H&E) and examined in a blinded manner by gastrointestinal pathologists (M.B.P. and M.K.W.). For DSS colitis, inflammation severity (0–3) and inflammation extent (0–3) were each multiplied by the percent involvement (1 = 0–25%, 2 = 25–50%, 3 = 50–75%, and 4 = 75–100%) and added together to yield the inflammation score (0–24); the parameter of crypt damage (0–4) was also multiplied by the percent involvement to yield an epithelial injury score (0–16). These scores were then added together to yield the histological injury score (0–40) (Singh et al., 2011, 2013; Coburn et al., 2012; Gobert et al., 2018). For *C. rodentium* colitis, the histologic injury score (0–21) was the sum of acute and chronic inflammation (0–3 for each) scores multiplied by extent of inflammation (0–3) plus the epithelial injury score (0–3), as described (Singh et al., 2011, 2016; Hardbower et al., 2017; Gobert et al., 2018).

### Quantification of Amino Acids

Frozen tissues were homogenized in 0.1 M trichloroacetic acid containing 10^−2^ M sodium acetate, 10^−4^ M EDTA, and 10.5% methanol (pH 3.8). After centrifugation at 10,000 *g* for 20 min, supernatants were used for protein assay using BCA and for LC/MS.

To prepare internal standards, 50 μl stock solutions of each amino acid (5 ng/μl) were diluted with 200 μl acetonitrile and 100 μl each of 500 mM Na_2_CO_3_ and 2% isotopically labeled benzoyl chloride (^13^C_6_-BZC) in acetonitrile. After 2 min, the reaction was stopped by the addition of 200 μl of 20% acetonitrile in water containing 3% sulfuric acid and 400 μl water. These solutions were diluted 100 X with 20% acetonitrile in water containing 3% sulfuric acid to make the working internal standard solution used in the sample analysis.

Cell extracts or cell supernatants (5 μl) were diluted in acetonitrile (20 μl), 500 mM Na_2_CO_3_ (10 μl), and 2% benzoyl chloride in acetonitrile (10 μl). After 2 min, the reaction was stopped by the addition of 20 μl of the internal standard solution and 40 μl water.

Liquid chromatography was performed on a 2 × 50 mm, 1.7 μm particle Acquity BEH C18 column (Waters Corporation) using a Waters Acquity UPLC. Mobile phase A was 0.15% aqueous formic acid and mobile phase B was acetonitrile. Samples were separated by a gradient of 98–5% of mobile phase A over 11 min at a flow rate of 600 μl/min prior to delivery to a SCIEX 6500+ QTrap mass spectrometer. The ratio of the peak height of the endogenous amino acids was compared to the peak height of the isotopically-labeled internal standards for quantitation. All data were analyzed using MultiQuant Software Version 3.0 (SCIEX).

### Quantification of Polyamines

The concentration in colon tissues of the three biogenic polyamines putrescine, spermidine, and spermine was determined by mass spectrometry as described (Hardbower et al., 2017; Gobert et al., 2018).

### Measurement of Cytokines and Chemokines

Colon tissues were lysed in Cell Lytic Mammalian Tissue Lysis Extraction Reagent (Sigma) and analyzed using the 32-analyte MILLIPLEX MAP Mouse Cytokine/Chemokine Magnetic Bead Panel (Millipore Sigma) on a FLEXMAP 3D instrument (Luminex) as reported (Coburn et al., 2018; Singh et al., 2018). Data were standardized to tissue protein concentrations measured by the BCA Protein Assay Kit (Pierce).

### Immunostaining

Immunofluorescent staining for NOS2, arginase-1 and the macrophage marker CD68 was performed on the colon tissues as described (Singh et al., 2018) using a rabbit polyclonal anti-NOS2 (Novus Biological; 1/100), a goat polyclonal anti-arginase-1 (Santa Cruz; 1/100), and a rabbit polyclonal anti-mouse CD68 Ab (Boster Biological, 1/100), respectively.

### Analysis of the Composition of Intestinal Microbiota

Colonic feces were collected from mice after euthanasia and were lysed using bead beating with the QIAGEN TissueLyser II. Genomic DNA was extracted using the QIAGEN Powersoil kit.

Amplicons in the V4 hypervariable region of 16S rRNA genes were amplified with MyTaq polymerase master mix (Bioline). In this step, amplicons of each sample were differently barcoded with primers 515F/806R (Kozich et al., 2013). ZymoBIOMICS (Zymo) positive controls and extraction and PCR negative controls were run alongside the samples. PCR products were run on 1.2% TAE agarose gels to verify reaction success. Amplicons were cleaned and normalized with the SequalPrep Normalization Plate Kit (Invitrogen). Samples were pooled and cleaned with 1X Ampure XP Beads (Beckman Coulter). Sequencing was performed on an Illumina MiSeq with 2 × 250 bp reads. Sequences were processed with mothur and aligned to the SILVA database release 123 and taxonomically classified with the Ribosomal Database Project classifier 11 (Pruesse et al., 2007; Cole et al., 2009). Non-bacterial sequences and chimeric sequences detected by UCHIME were removed. Operational Taxonomic Unit (OTU) clustering was performed with VSEARCH, using abundance-based greedy clustering (Rognes et al., 2016).

### Statistics

All the data shown represent the mean ± SEM. Data that were not normally distributed according to the D'Agostino & Pearson normality test were log transformed. Statistics for the Luminex analysis was performed using the two-stage-step-up method of Benjamini, Krieger, and Yekutieli that correct for multiple comparisons by controlling the False Discovery rate (*Q* < 0.05). The relative abundance of the phyla, families, and genus in Arg^0^, Arg^NL^, and Arg^HIGH^ groups was analyzed by the Kruskal-Wallis test and the multiple comparison testing was performed using the Uncorrected Dunn's test. Microbiome richness was estimated with the Chao1 and ACE indices, and alpha-diversity was estimated with Hill numbers N1 and N2 which are, respectively, the exponential of the Shannon index and inverted Simpson index. A beta diversity dissimilarity matrix (Bray-Curtis) was computed over the multiple rarefactions and the permutation-based ANOVA (PerMANOVA) was used to test for associations between microbial profiles and arginine treatment. Rarefaction followed by richness, alpha-diversity, and beta-diversity calculations were repeated 400 times, and the results were averaged. Detailed methods of estimating microbiome richness and alpha- and beta-diversity has been previously described (Shilts et al., 2016). In all other experiments, the Student's *t* test or ANOVA with the Tukey test were used to determine significant differences between two groups or to analyze significant differences among multiple test groups, respectively.

## Results

### Arg Supplementation Improves DSS-Induced Colitis

**Figure 1:**
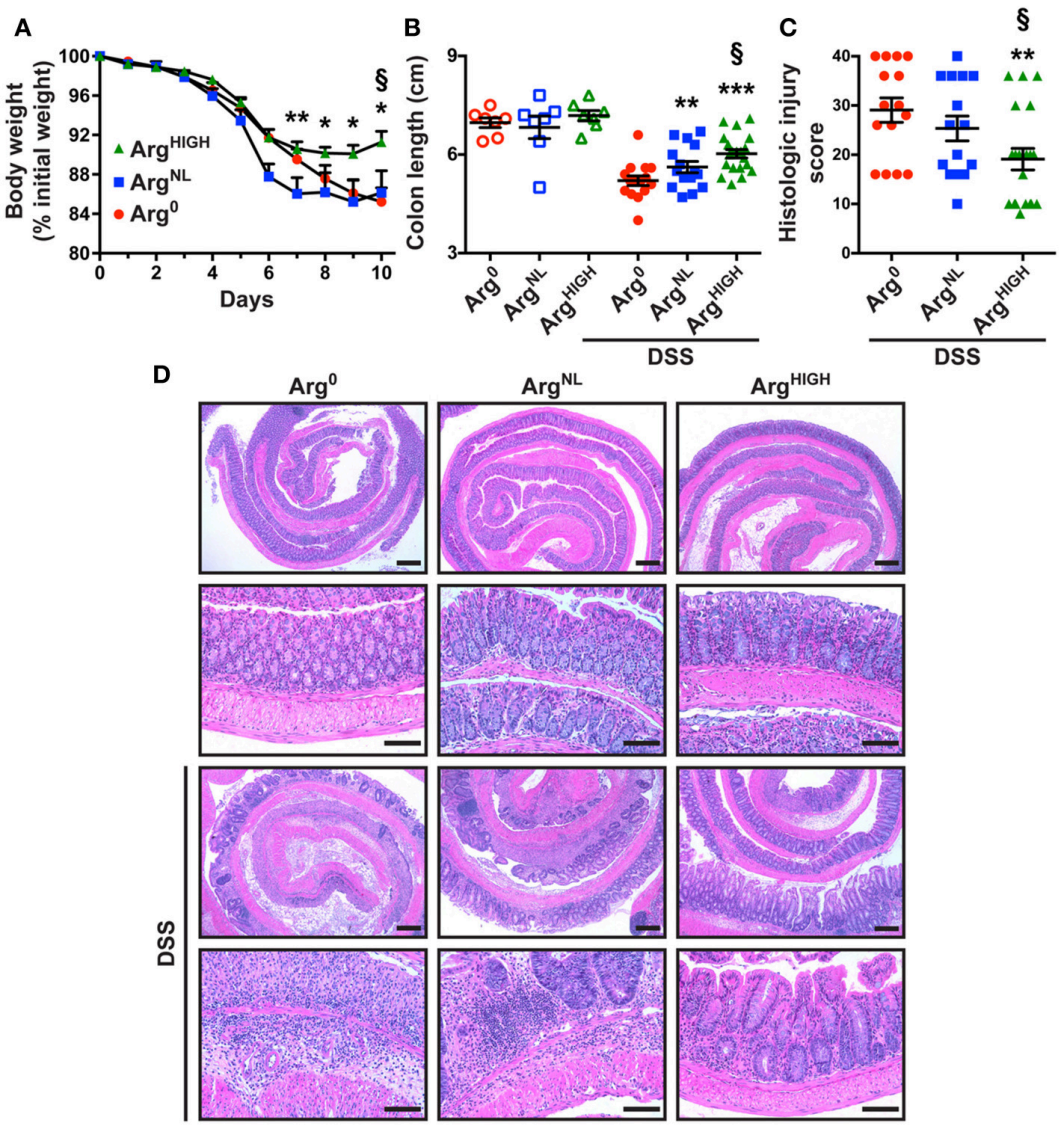
Effect of Arg on DSS colitis. C57BL/6 mice were treated with 2.5% DSS for 5 days and then kept for 5 more days under conditions of Arg^0^, Arg^NL^, or Arg^HIGH^ diets. **(A)** Body weights were monitored daily and are presented as percentage of initial body weight. **(B)** Colons were harvested and measured. **(C,D)** Colons were Swiss-rolled and stained with H&E **(D)** and scored for histologic injury **(C)**; the histologic injury score of mice without DSS was 0. Scale bar, 50 μm. In all panels, *P < 0.05, **P < 0.01, and ***P < 0.001 compared to DSS-treated mice on the Arg^0^ diet; §*P* < 0.05 vs. DSS-treated mice on the Arg^NL^ diet, by ANOVA with the Tukey test.

To assess the effect of Arg dietary regimens on colonic inflammation, we fed mice with Arg^0^, Arg^NL^, or Arg^HIGH^ diets during the recovery period, after DSS treatment. Mice began losing weight during the 5 days of DSS treatment (Figure 1A). However, body weight loss was significantly improved with the Arg^HIGH^ diet compared to animals on the Arg^0^ or Arg^NL^ diets. Mice on the Arg^0^, Arg^NL^, or Arg^HIGH^ regimens without DSS treatment did not have differences in body weight throughout the course of the experiments (Figure S1). The shortening of the colon, which is an indicator of disease severity in DSS-treated mice (Singh et al., 2018), was improved in mice on the Arg^NL^ or Arg^HIGH^ diet compared to those fed the Arg^0^ diet, and was also improved on the Arg^HIGH^ diet when compared to the Arg^NL^ diet (Figure 1B). Lastly, we observed decreased overall histological injury scores in DSS-treated mice receiving the Arg^HIGH^ diet compared to mice with Arg^0^ or Arg^NL^ diets (Figure 1C). H&E-stained sections of the distal colon of control mice showed no inflammation and no change between the animals on the different Arg diets (Figure 1D). The colon of DSS-treated mice exhibited severe inflammatory infiltrates (neutrophils and lymphocytes), crypt loss, and ulceration (Figure 1D). All these parameters were improved with the Arg^HIGH^ diet (Figure 1D).

### Amino Acid Profiling in Response to the Different Arg Diets

**Figure 2:**
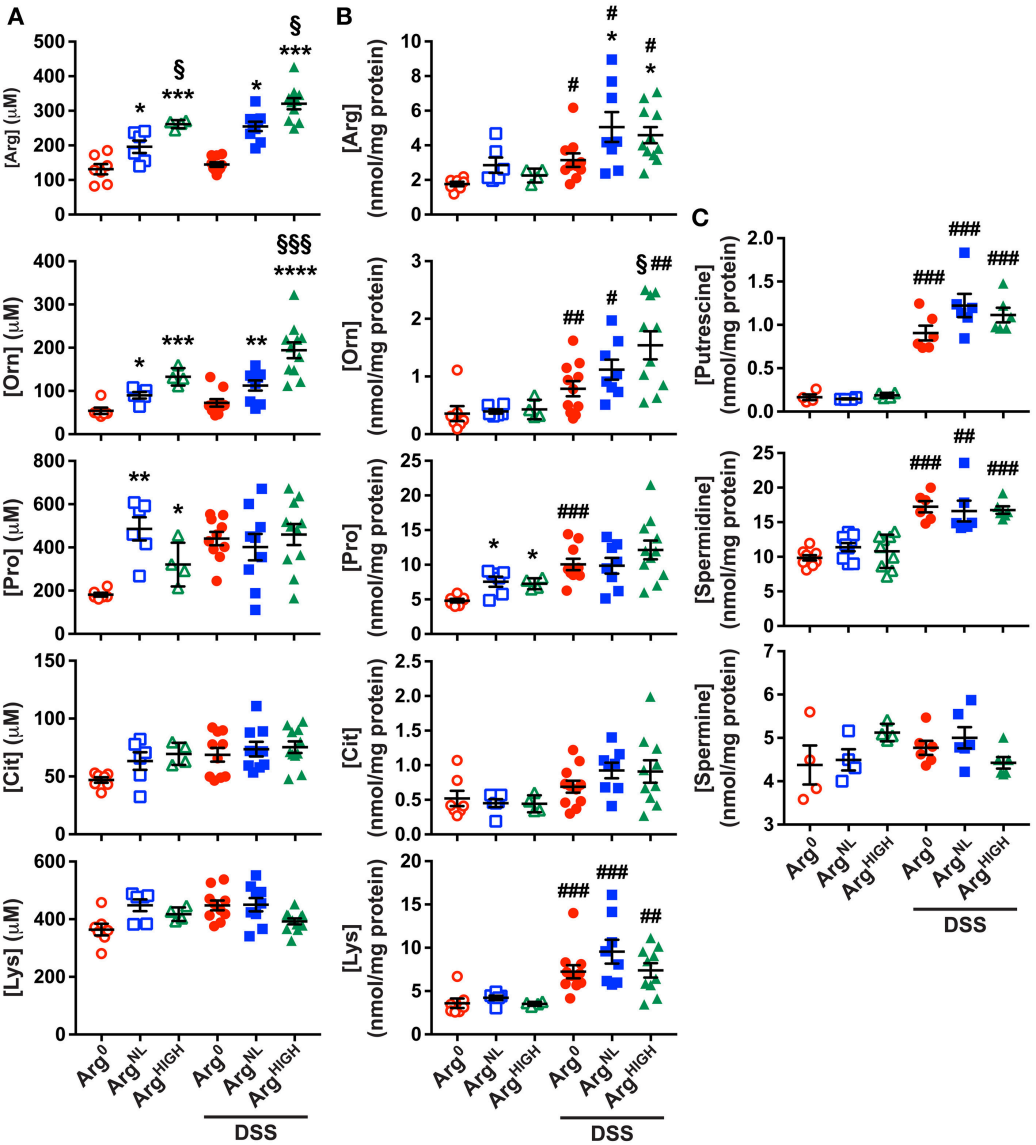
Amino acid and polyamine profiles during DSS colitis. **(A,B)** Concentration of amino acids in the serum **(A)** and in the colon **(B)** of mice. **(C)** The concentration of putrescine, spermidine, and spermine was determined by LC/MS in the colonic tissues. In all panels, #*P* < 0.05, ##*P* < 0.01, and ###*P* < 0.001 denote significant differences compared to animals not treated with DSS; *P < 0.05, **P < 0.01, ***P < 0.001, and ****P < 0.0001 compared to DSS-treated mice on the Arg^0^ diet; §*P* < 0.05 and §§§*P* < 0.001 vs. DSS-treated mice on the Arg^NL^ diet, by ANOVA with the Tukey test.

After 5 days of DSS followed by 5 days of the special diet regimens, the concentrations of the amino acids related to Arg metabolism (Morris, 2004) were determined in the serum and the colon by mass spectrometry. We analyzed (1) Arg; (2) Orn that is principally generated from Arg by the enzyme arginase; (3) Pro that results from the conversion of Orn by ornithine aminotransferase; (4) L-citrulline (Cit), the product of the conversion of Arg by NOS; and (5) L-lysine (Lys), an amino acid that competes with arginine for the same transport system. In the serum, the concentration of these amino acids was not affected by the treatment of the mice with DSS (Figure 2A); however, Arg, Orn, Pro, and Lys concentrations in the colon were significantly increased in animals with DSS colitis compared to control mice (Figure 2B). As expected, serum Arg was increased in mice on the Arg^NL^ diet compared to animals receiving Arg^0^ diet, and was further enhanced on the Arg^HIGH^ diet (Figure 2A); this was observed in both untreated mice and DSS-treated animals (Figure 2A). In the colon, the increase in Arg concentration was observed only in animals that were given DSS (Figure 2B). Orn and Pro were also enhanced in the serum of Arg^HIGH^-treated mice (Figure 2A); but only Orn concentration was significantly higher in the colonic tissue of mice treated with DSS and fed the Arg^HIGH^ diet compared to the Arg^0^ diet (Figure 2B). In contrast, Cit and Lys concentrations were not significantly affected by the various Arg diets (Figures 2A,B).

Because (1) Orn concentration was increased in DSS-treated mice and in mice fed the Arg^HIGH^ diet and (2) Orn is the substrate of ODC that synthesizes the first polyamine putrescine, we determined polyamine concentrations in the colonic tissues. Levels of putrescine and spermidine, but not spermine, were increased in colitic mice compared to control animals (Figure 2C), but the different Arg regimens had no effect on tissue polyamine content (Figure 2C).

### Supplementation of the Diet With Orn or Pro Does Not Protect From DSS Colitis

Since we found that Orn and Pro concentrations were increased in the serum and/or colon of DSS-treated mice fed the Arg^HIGH^ diet, we reasoned that Arg might exert its protective effect in DSS colitis through the synthesis of these two amino acids. To test this hypothesis, mice were treated with DSS and then fed Orn^NL^, Orn^HIGH^, Pro^NL^, or Pro^HIGH^ diets (Table 1). The loss of body weight (Figure S2A), the reduction of colon length (Figure S2B), and the histological damage (Figure S2C) induced by DSS was not improved in mice that were given Orn^HIGH^ or Pro^HIGH^ diets compared to animals receiving Orn^NL^ or Pro^NL^ regimens, respectively. These results imply that Arg protects mice independently of Orn or Pro synthesis.

### Arg Supplementation Improves *C. rodentium*-Induced Colitis

**Figure 3:**
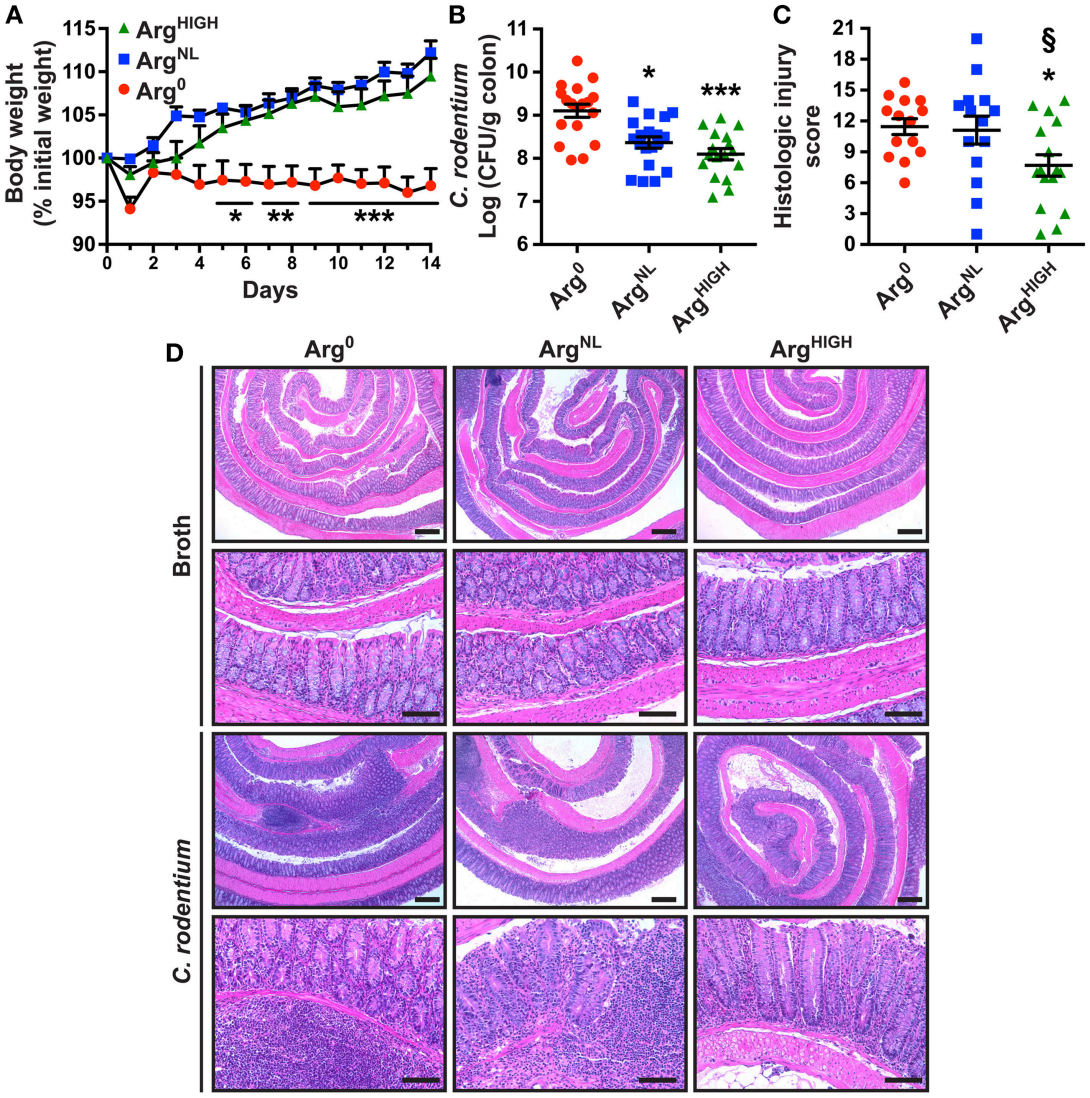
Outcome of *C*. *rodentium* infection in response to Arg. C57BL/6 mice were infected with *C*. *rodentium*. **(A)** Body weights were measured every day and are presented as percentage of initial body weight. **(B)** After 14 days, *C. rodentium* colonization in the colon was assessed by plating serial dilutions. **(C,D)** Colons were Swiss-rolled and stained with H&E **(D)** and scored for histologic injury **(C)**. Scale bar, 50 μm. In all panels, *P < 0.05, **P < 0.01, and ***P < 0.001 compared to DSS-treated mice on the Arg^0^ diet; §*P* < 0.05 vs. DSS-treated mice on the Arg^NL^ diet, by ANOVA with the Tukey test.

To determine whether Arg is protective in another model of colitis, we used the pathogen *C. rodentium* to infect C57BL/6 mice. Animals infected with *C. rodentium* on the Arg^0^ diet did not gain weight during the 14 days of infection, whereas the mice fed Arg^NL^ or Arg^HIGH^ diets increased their body weight to the same degree as uninfected animals (Figure 3A and Figure S1). *C. rodentium* burden was significantly less in mice receiving Arg^NL^ or Arg^HIGH^ diets compared with mice on the Arg^0^ diet (Figure 3B). Moreover, the histologic damage was attenuated in infected mice on the Arg^HIGH^ diet compared to *C. rodentium*-infected mice on the two other regimens (Figure 3C). H&E staining of the colons of mice infected with *C. rodentium* exhibited an effacement of the brush border, hyperplasia, severe mucosal inflammation, and submucosal edema compared to uninfected mice (Figure 3D). However, these abnormalities were less present in animals that were given the Arg^HIGH^ diet (Figure 3D).

### Alteration of the Mucosal Immune Response by Arg

Next, we investigated the levels of chemokines/cytokines in the colon tissues in response to colitis and the different Arg diets. As shown in Table 2, we found that there were 15 analytes upregulated in both DSS and *C. rodentium* colitis, including innate immune cell-associated cytokines (G-CSF, IL-1β, TNF-α, LIF, and IL-6), chemokines (CCL2, CCL5, CCL11, CXCL2, CXCL8, CXCL9, MIP-1α, and MIP-1β), and Th1 (IFN-γ) and Th17 (IL-17) cytokines (Table 2). Among these analytes upregulated in colitis, the levels of G-CSF, CCL2, and CXCL2 were significantly reduced in mice on the Arg^HIGH^ diet in the DSS model (Table 2). In addition, the concentrations of G-CSF, IL-1β, CCL2, and IL-6 in the colon of mice infected with *C. rodentium* and receiving the Arg^NL^ diet or the Arg^HIGH^ diet were also significantly reduced compared to infected animals that were given the Arg^0^ diet (Table 2). These data suggest that the innate response of the colonic mucosa is the main component of the immune response dampened by Arg supplementation.

**Table 2:** Concentration of cytokines and chemokines in the colon^a^.

	**Control**			**DSS**			***C. rodentium***		
	**Arg^**0**^**	**Arg^**NL**^**	**Arg^**HIGH**^**	**Arg^**0**^**	**Arg^**NL**^**	**Arg^**HIGH**^**	**Arg^**0**^**	**Arg^**NL**^**	**Arg^**HIGH**^**
G-CSF	0.7 ± 0.1	0.6 ± 0.2	0.7 ± 0.1	592.3 ± 185^*^	938.2 ± 333.2^**^	295.5 ± 128.2^§^	63.1 ± 14.4^****^	10.2 ± 4.1^***^^§§§^	27.2 ± 11.3^****^^§^
GMCSF	0.2 ± 0.1	0.2 ± 0.1	0.2 ± 0.1	0.6 ± 0.3	0.5 ± 0.3	0.1 ± 0.1	3.7 ± 1.0	1.2 ± 0.6	1.0 ± 0.3
MCSF	0.4 ± 0.1	0.3 ± 0.1	0.2 ± 0.1	1.1 ± 0.5	1.0 ± 0.4	1.0 ± 0.3	0.2 ± 0.1	0.2 ± 0.1	0.2 ± 0.1
IL-1α	63.5 ± 18.9	96.3 ± 13.9	81.4 ± 9.6	69.5 ± 4.2	82.7 ± 7.7	49.6 ± 11.5	57.2 ± 7.4	94.8 ± 10.1	63.1 ± 9.3
IL-1β	0.9 ± 0.1	0.7 ± 0.1	2.1 ± 1.0	28.21 ± 17.9^**^	13.1 ± 4.8^***^	11.9 ± 5.6	93.3 ± 38.9^**^	22.6 ± 11.1^*^^§^	11.1 ± 3.0^*^^§^
IL-2	2.8 ± 0.5	2.8 ± 0.5	1.8 ± 0.3	2.3 ± 0.5	2.7 ± 0.6	1.2 ± 0.3	1.2 ± 0.1	2.1 ± 0.1	1.5 ± 0.2
IL-3	0.1 ± 0.1	0.1 ± 0.1	0.1 ± 0.1	0.1 ± 0.1	0.1 ± 0.1	0.1 ± 0.1	0.1 ± 0.1	0.1 ± 0.1	0.1 ± 0.1
IL-4	0.5 ± 0.2	0.4 ± 0.1	0.3 ± 0.1	0.4 ± 0.1	0.4 ± 0.1	0.3 ± 0.1	0.4 ± 0.1	0.4 ± 0.1	0.3 ± 0.1
IL-5	0.4 ± 0.1	0.1 ± 0.1	0.2 ± 0.1	0.4 ± 0.1	0.3 ± 0.1	0.2 ± 0.1	0.9 ± 0.4	0.2 ± 0.1	0.3 ± 0.1
IL-6	0.3 ± 0.1	0.3 ± 0.1	0.4 ± 0.1	78.5 ± 21.3^****^	165.5 ± 64.7^****^	68.1 ± 28.1^****^^§^	75.6 ± 31.2^****^	4.8 ± 2.0^**^^§§§^	32.6 ± 15.1^**^^§^
IL-7	1.5 ± 0.2	2.5 ± 0.3	2.8 ± 0.2	2.2 ± 0.5	2.1 ± 0.5	3.3 ± 0.4	2.5 ± 0.2	2.5 ± 0.2	3.6 ± 0.4
IL-9	634.2 ± 76.7	400.6 ± 54.7	290.1 ± 30.6	741.5 ± 90.9	898.3 ± 250.8	400.5 ± 29.9	296.3 ± 24.4	362.5 ± 21.2	304.1 ± 36.7
IL-10	1.8 ± 0.2	1.4 ± 0.3	1.9 ± 0.5	5.3 ± 1.0	5.8 ± 1.0	2.6 ± 0.6	2.0 ± 0.2	2.1 ± 0.3	2.1 ± 0.3
IL-12p40	0.8 ± 0.1	0.7 ± 0.1	0.5 ± 0.1	0.7 ± 0.1	0.6 ± 0.1	0.5 ± 0.1	0.8 ± 0.1	0.7 ± 0.1	0.5 ± 0.1
IL-12p70	1.1 ± 0.1	0.9 ± 0.1	0.7 ± 0.1	0.8 ± 0.1	0.9 ± 0.1	0.6 ± 0.1	0.7 ± 0.1	0.8 ± 0.1	0.7 ± 0.1
IL-13	1.4 ± 0.1	1.2 ± 0.1	0.9 ± 0.1	6.3 ± 4.4	9.1 ± 4.0	11.7 ± 4.8	5.4 ± 2.0	4.7 ± 1.6	2.9 ± 0.7
IL-15	1.6 ± 0.1	1.7 ± 0.3	2.2 ± 0.5	4.1 ± 0.5	3.5 ± 1.1	0.8 ± 0.5	3.4 ± 1.1	2.8 ± 0.5	4.9 ± 1.6
IL-17	0.6 ± 0.2	1.1 ± 0.1	1.3 ± 0.3	8.8 ± 1.8^****^	35.0 ± 10.9^****^	10.3 ± 5.7^*^	13.4 ± 2.5^****^	12.02 ± 2.7^***^	6.9 ± 0.9^***^
LIX	0.9 ± 0.1	0.7 ± 0.1	0.6 ± 0.1	1.4 ± 0.7	20.1 ± 14.6	3.5 ± 3.0	7.9 ± 2.1	9.9 ± 8.1	8.2 ± 6.7
LIF	0.8 ± 0.2	0.7 ± 0.1	0.7 ± 0.1	6.7 ± 1.3^****^	6.5 ± 1.2^****^	6.1 ± 1.5^****^	28.5 ± 3.3^****^	19.0 ± 2.4^****^	21.0 ± 4.1^****^
CXCL9	70.5 ± 29.2	436.6 ± 169.4	1380 ± 905	2840 ± 704.5^****^	6275 ± 1479^****^	5070 ± 640.5^****^	7574 ± 642.1^****^	7184 ± 1559^****^	7371 ± 655.2^****^
CXCL8	9.4 ± 2.7	14.5 ± 1.5	14.1 ± 0.4	152.6 ± 29.6^****^	293.6 ± 100.6^***^	112.1 ± 40.2^**^	123.7 ± 16.6^****^	80.4 ± 23.9^***^	99.2 ± 23.6^***^
CCL2	5.2 ± 0.3	4.3 ± 0.2^*^	3.4 ± 0.2	16.2 ± 7.2^*^	12.8 ± 5.8	7.7 ± 4.6	32.9 ± 8.2^***^	4.7 ± 0.9^§§§^	17.9 ± 8.7^§^
MIP-1α	3.9 ± 1.1	4.7 ± 1.3	2.8 ± 0.7	28.9 ± 5.7^**^	27.6 ± 8.7^**^	16.2 ± 6.4^*^	31.4 ± 4.0^**^	30.6 ± 7.3^***^	21.5 ± 4.1^**^
MIP-1β	1.8 ± 0.1	1.4 ± 0.1	1.2 ± 0.1	45.6 ± 6.7^****^	123.6 ± 69.9^****^	57.5 ± 23.1^****^	50.9 ± 8.2^****^	62.4 ± 11.2^****^	42.2 ± 7.1^****^
CXCL2	11.7 ± 0.6	8.3 ± 0.4	7.1 ± 0.8	445.7 ± 165.2^***^	700.6 ± 433.9^***^	322.2 ± 207.3^*^^§^	214.0 ± 47.1^***^	107.0 ± 31.2^**^	123.2 ± 51.0^**^
CCL5	4.1 ± 0.3	4.1 ± 0.3	5.5 ± 0.6	31.8 ± 5.8^****^	45.9 ± 10.1^****^	20.1 ± 4.2^***^	25.3 ± 5.9^***^	21.2 ± 3.8^**^	29.7 ± 9.1^***^
VEGF	194.1 ± 22.2	118.4 ± 99.5	83.9 ± 10.7	66.2 ± 8.2	66.2 ± 9.6	59.1 ± 15.8	98.2 ± 8.2	66.6 ± 12.3	69.6 ± 8.2
IP10	46.9 ± 2.2	58.1 ± 6.0	84.8 ± 24.6	208.4 ± 79.6	2238 ± 1100^**^	838.4 ± 300.5^*^	6902 ± 657.6	4561 ± 1322	4516 ± 1239
IFN-γ	1.9 ± 1.2	3.5 ± 0.6	4.3 ± 0.7	5.1 ± 0.7^*^	19.7 ± 6.8	3.1 ± 0.6	70.4 ± 13.0^****^	29.1 ± 9.4^*^	33.4 ± 10.7^*^
TNF-α	0.4 ± 0.1	0.3 ± 0.1	0.2 ± 0.1	3.2 ± 1.0^**^	3.7 ± 1.2^****^	3.3 ± 1.3^***^	9.1 ± 1.0^****^	4.9 ± 1.3^***^	6.7 ± 1.8^****^
CCL11	295 ± 27.8	370.6 ± 53.4	594.2 ± 106	1379 ± 274.3^****^	1217 ± 80.2^***^	1185 ± 273.2^*^	1402 ± 272^***^	893.9 ± 187^**^	924.3 ± 140.9

a *Values are expressed in pg/mg protein*.

* *P < 0.05*,

** *P < 0.01*,

*** *P < 0.001*,

**** *P < 0.0001 vs. Arg-matched controls*.

§ *P < 0.05*,

§§§ *P < 0.001 compared to the DSS-treated mice or C. rodentium-infected mice in the Arg^0^ group; n = 4 mice in the Control group and 6 mice for DSS and C. rodentium groups*.

**Figure 4:**
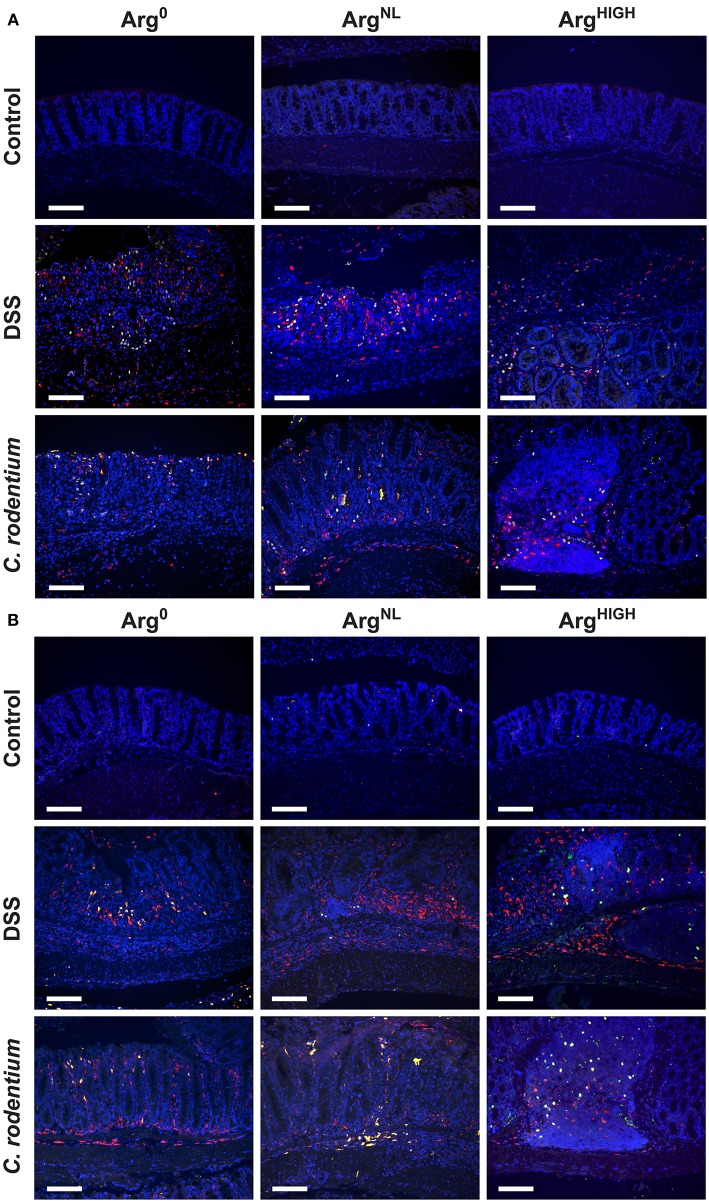
Regulation of NOS2 and arginase-1. NOS2, arginase-1, and CD68 were immunodetected in colonic tissues from C57BL/6 mice ± DSS or *C. rodentium* ± Arg^0^, Arg^NL^, or Arg^HIGH^ diets. In each panel, CD68 is depicted in red, NOS2 **(A)** or arginase-1 **(B)** in green, and the nuclei in blue; CD68^+^NOS2^+^ and CD68^+^ARG1^+^ cells are shown in yellow. The data shown are representative photomicrographs of at least 3 animals per condition. Scale bar, 50 μm.

We then assessed the protein expression of NOS2 and arginase-1 in colonic macrophages. These two enzymes use Arg as a substrate and are the prototypical markers of M1 and M2 macrophages, respectively. Overall, we found increased NOS2^+^/CD68^+^ cells in the colonic mucosa of mice treated with DSS or infected with *C. rodentium* compared to control animals (Figure 4A). In both models, NOS2 protein expression was reduced in mice treated with the Arg^HIGH^ diet (Figure 4A). Arginase-1 was also induced in mice with DSS or *C. rodentium* colitis compared to untreated animals, but its expression was not affected by Arg treatment (Figure 4B).

### The AIN-76A Diet Disturbs the Composition of the Gut Microbiota

**Figure 5:**
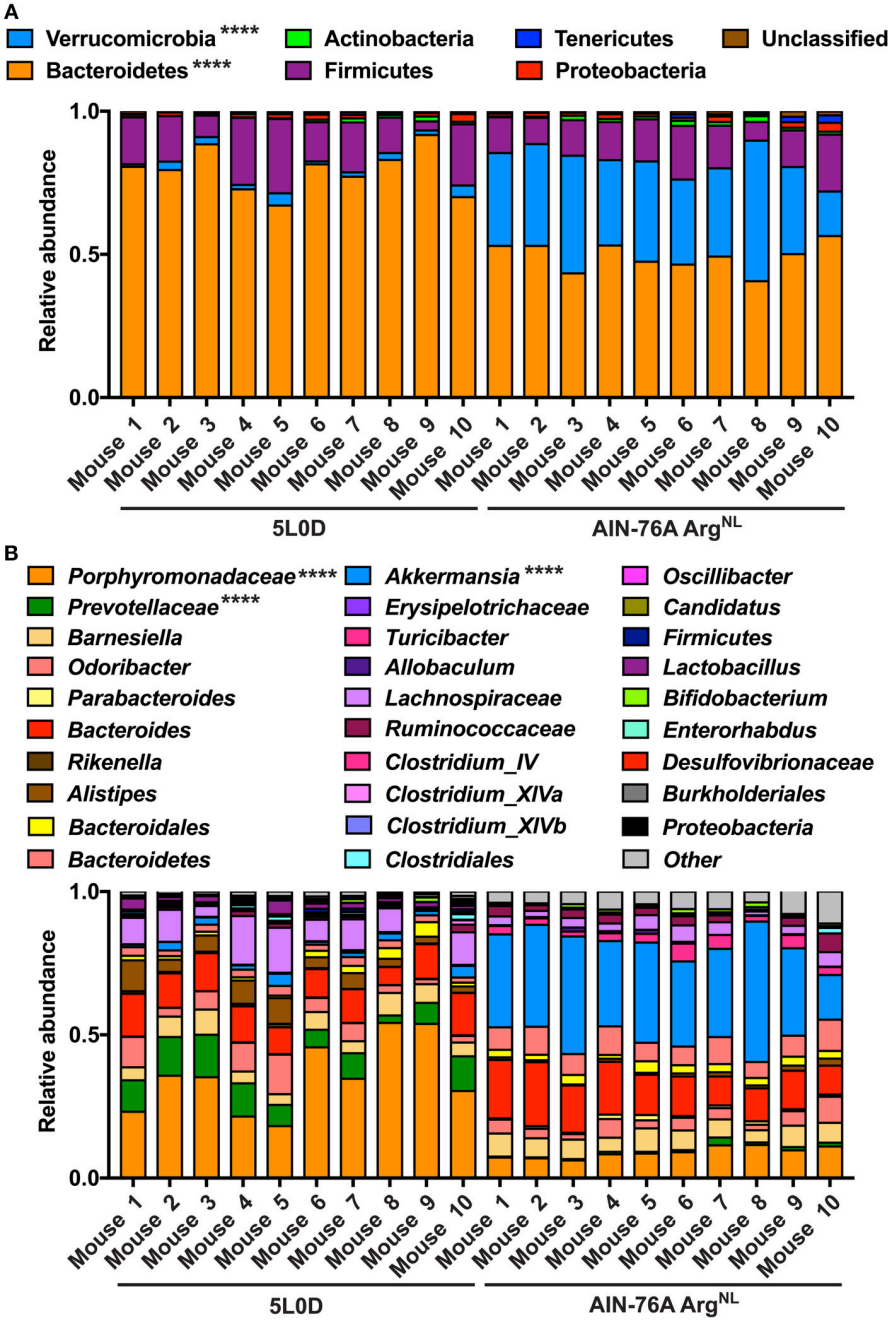
Analysis of the microbiome during diet change. C57BL/6 mice on the 5L0D diet were switched to the AIN-76A Arg^NL^ regimen for 7 days. Feces were collected before the diet change (5L0D) and after the period on the AIN-76A Arg^NL^ diet (AIN-76A). The percentage of each phylum **(A)** and genus **(B)** is shown. Mice 1–5 and 6–10 were distributed in two different cages. ****P < 0.0001 denote significant differences between the 5L0D and AIN-76A groups, determined by the Student's paired *t* test.

Because mice were maintained under the special AIN-76A diet during the experiments, we first analyzed the composition of their microbiota under this regimen. The feces of mice (6–8 weeks old) fed the regular 5L0D chow were first collected; animals were then given the AIN-76A Arg^NL^ diet for 7 days and the feces of these mice were also collected. Sequencing of V4 region of 16S rRNA showed that the fecal microbiota of the mice that were given the 5L0D regimen was dominated by the Bacteroidetes and Firmicutes phyla (Figure 5A). However, the prevalence of the Bacteroidetes phylum was markedly reduced when mice were fed the AIN-76A diet, whereas the abundance of Verrucomicrobia was significantly increased (Figure 5A). The relative abundance of Firmicutes, Actinobacteria, Tenericutes, and Proteobacteria was not affected by the change in diet. This profound modification of the composition of the gut microbiota was also confirmed at the family and genus level: The prevalence of *Porphyromonadaceae* and *Prevotellaceae*, which belongs to the Bacteroidetes phylum, was significantly reduced (Figure 5B); in contrast, the genus *Akkermansia* (Derrien et al., 2004), which is the sole cultivated representative of the Verrucomicrobia phylum, was more abundant in mice receiving the AIN-76A diet compared to those fed with the 5L0D chow (Figure 5B).

### Arg Supplementation Affects the Gut Microbiome Diversity

**Figure 6:**
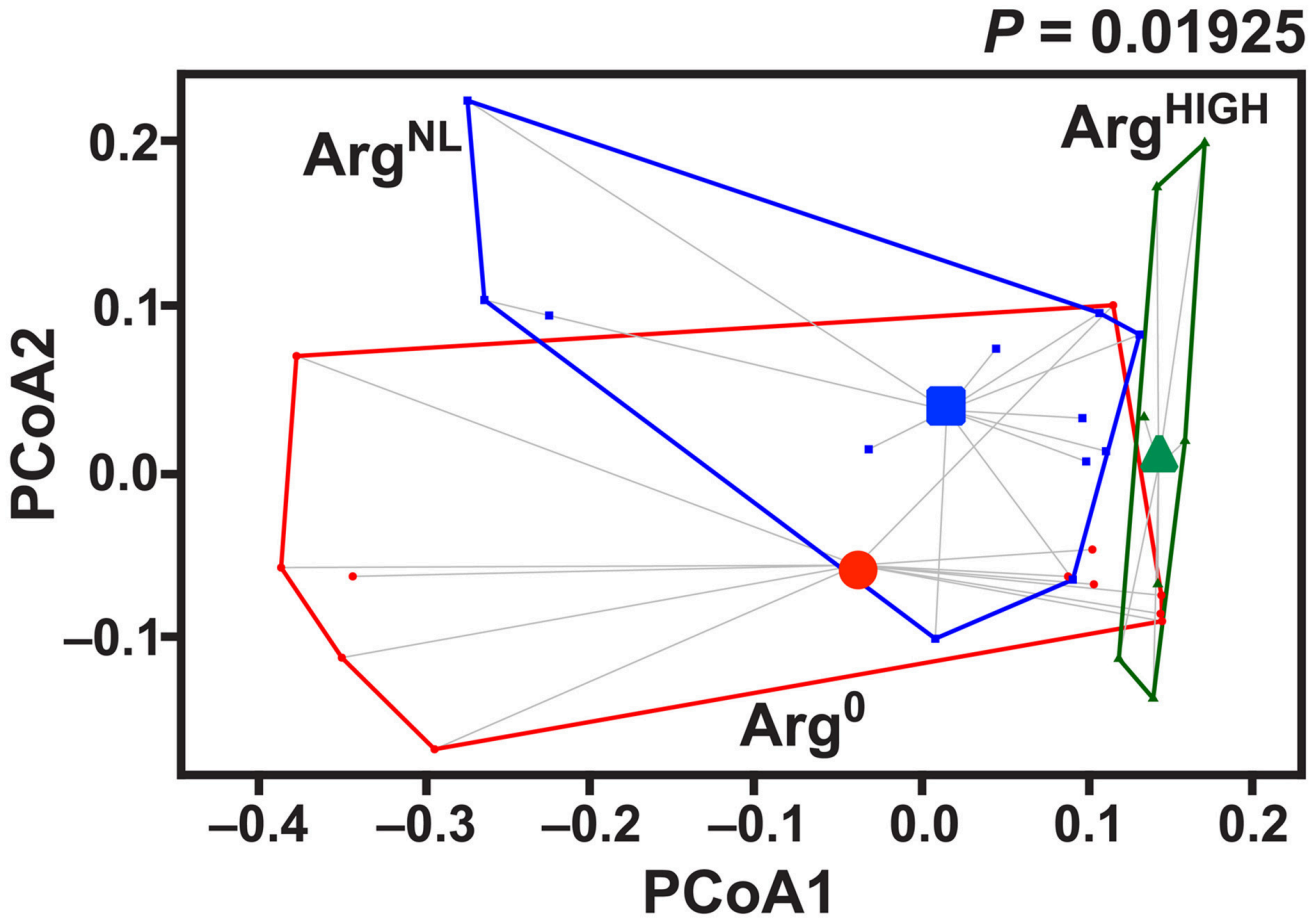
Effect of Arg on gut microbiota structure. Mice were fed for 7 days with AIN-76A Arg^NL^ diet and then with the Arg^0^, Arg^NL^, or Arg^HIGH^ regimens for 7 more days. After sequencing, the PCoA plot of microbial species abundance using the different Arg diet as grouping variable, based on the Bray-Curtis distances, was established. The significance between the groups was determined by PerMANOVA; *n* = 7–12 mice per group.

It has been reported that the composition of the microbiota can modulate the development of colitis (Gobert et al., 2016; Johnston et al., 2018). We thus analyzed the composition of the intestinal microbiota in mice fed for 5 days with Arg^0^, Arg^NL^, or Arg^HIGH^ diets. Thirty-one mouse fecal samples were retained for microbial analysis. Sequencing analysis performed on fecal DNA generated an average of 28,190 high-quality, taxonomically classifiable 16S rRNA gene sequences with mean read lengths of 243 nt. Feeding the animals the diets containing various amount of Arg had no effect on the total number of bacteria in the colonic feces (Table 3). There was also no significant difference in estimated OTU richness between the three groups, as determined by the Chao1 and Ace metrics (Table 3). However, based on the one-way ANOVA test of the Shannon diversity index and the Simpson index, we found that the diversity of the intestinal microbiota was significantly enhanced in mice that were given the Arg^HIGH^ diet compared to the two other regimens (Table 3), suggesting that the relative abundance of the gut microbiota bacterial species is enhanced by Arg supplementation. Visualization of the beta diversity distance matrix using principal coordinate analysis (PCoA) showed that there were significant structural differences in the gut bacterial community between mice fed Arg^0^, Arg^NL^, and Arg^HIGH^ diets (Figure 6).

**Table 3:** Effect of Arg diets on alpha diversity of gut microbiota.

	**Arg^**0**^**	**Arg^**NL**^**	**Arg^**HIGH**^**
Bacterial abundance^a^	12.9 ± 0.1	13.2 ± 0.1	13.1 ± 0.2
**RICHNESS ESTIMATORS**			
Chao1	209.2 ± 12.4	232.9 ± 7.0	222.1 ± 13.8
Ace	208.0 ± 11.6	231.9 ± 7.0	222.3 ± 12.3
**DIVERSITY ESTIMATORS**			
Shannon	18.1 ± 3.8	17.2 ± 1.5	28.2 ± 3.7^*^^,^ ^§^
Simpson	4.9 ± 0.7	5.7 ± 0.5	13.2 ± 1.9^****^^,^ ^§§§^

a *Values expressed as Log (number of total bacteria/g feces); n = 5 mice*.

* *P < 0.05*,

**** P < 0.0001 compared to Arg^0^ and

§ *P < 0.05*,

§§§ *P < 0.001 compared to Arg^NL^ using Tukey's multiple comparisons test; n = 7–12 mice*.

**Figure 7:**
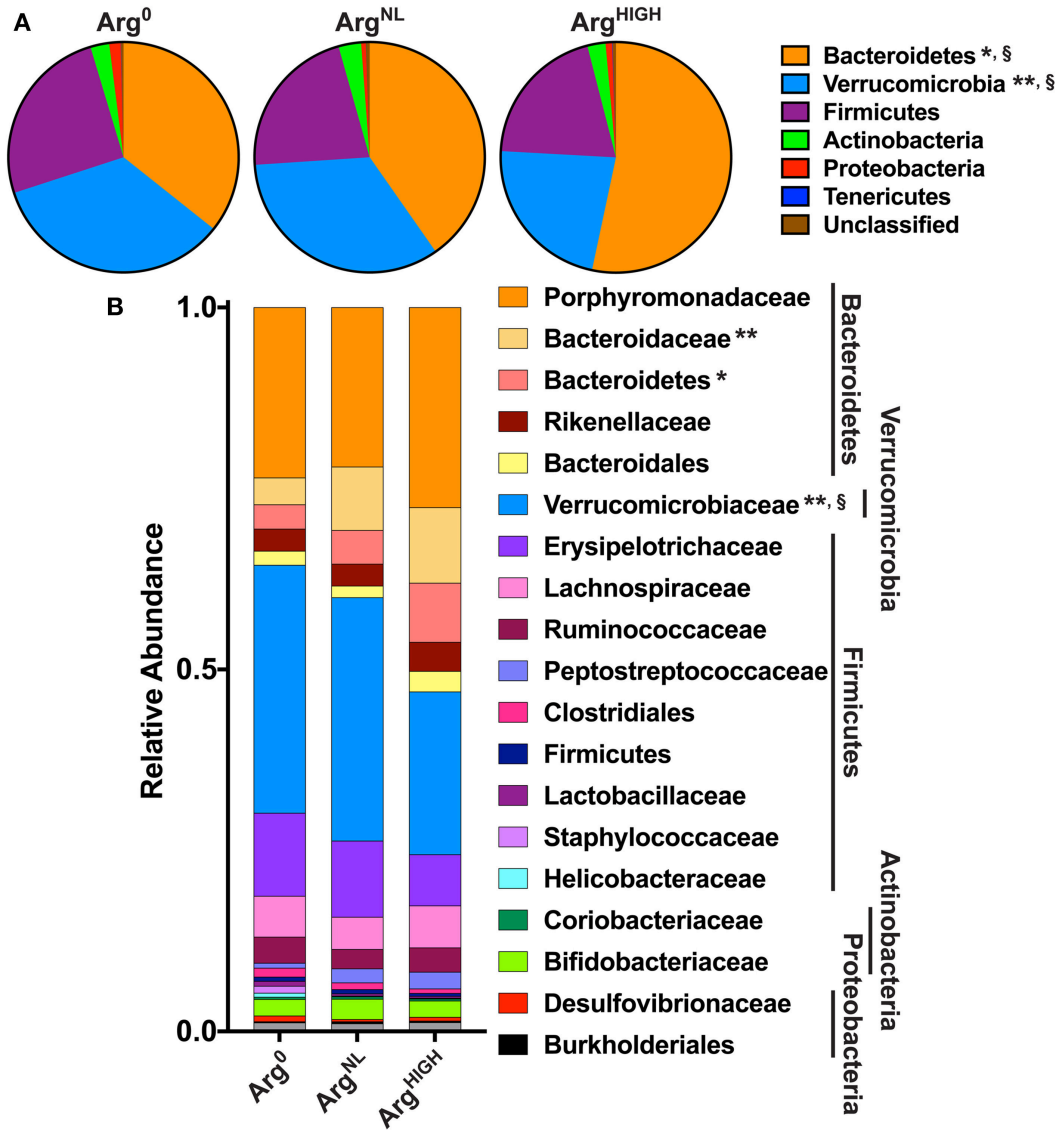
Analysis of the gut bacterial community composition. Variation in bacterial community composition at phylum **(A)** and family **(B)** levels is expressed as a percentage of the total community. *P < 0.05, **P < 0.01 denotes significant difference vs. Arg^0^ group; §*P* < 0.05, compared to the Arg^NL^ group; statistics were performed using ANOVA with the Tukey test.

At the phylum level, the fecal microbiota of the mice used in these experiments was dominated by Bacteroidetes, Verrucomicrobia, and Firmicutes. However, animals treated with the Arg^HIGH^ diet harbored significantly more Bacteroidetes and less Verrucomicrobia than mice on Arg^0^ or Arg^NL^ diets (Figure 7A). This was confirmed at the family level, since mice on the Arg^HIGH^ diet harbored more Bacteroidaceae and Bacteroidetes, and less Verrucomicrobiaceae than animals on Arg^0^ or Arg^NL^ diets (Figure 7B).

**Figure 8:**
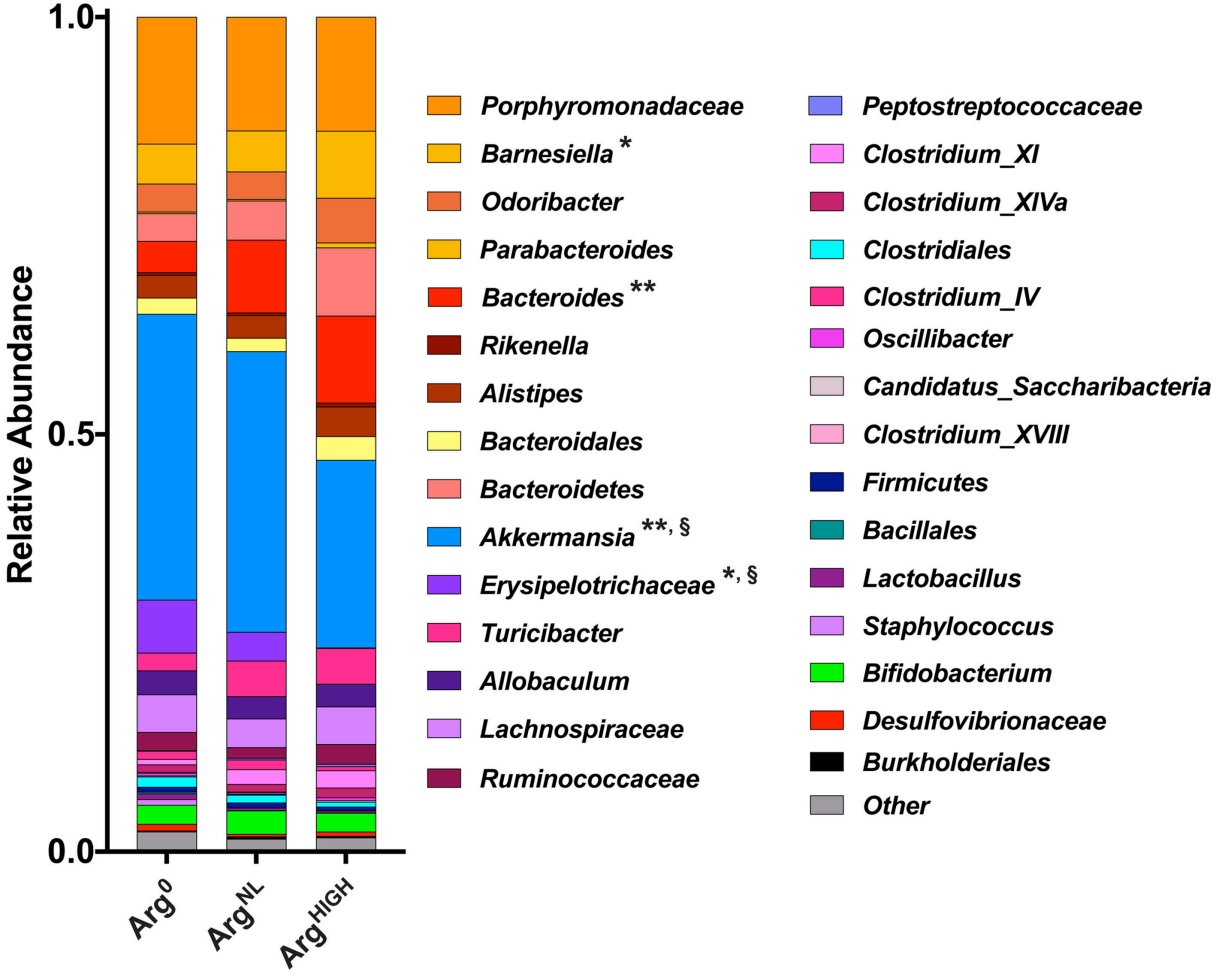
Composition of the gut microbiota at the genus level. Variation in bacterial community composition at genus levels expressed as a percentage of the total community. *P < 0.05, **P < 0.01 denotes significant difference vs. Arg^0^ group; §*P* < 0.05, compared to the Arg^NL^ group; statistics were performed using ANOVA with the Tukey test.

At the genus level, the most abundant bacterial genera detected were *Porphyromonadaceae, Barnesiella, Odoribacter, Bacteroides*, and *Akkermansia* (Figure 8), with *Barnesiella* and *Bacteroides* being more abundant and *Akkermansia* less present in animals on the Arg^HIGH^ diet (Figure 8).

## Discussion

Complementary and alternative medicines and/or adapted diets may supplement conventional therapies and improve symptoms of IBD patients. Herbal medicine or mind/body interventions have been tested in these patients and appear to display some benefits (Langhorst et al., 2015), but further strategies demonstrating a physiological impact are needed. In this study, we showed that the treatment of mice with an Arg-rich diet is protective in both DSS and *C. rodentium* colitis. The beneficial role of Arg supplementation in the drinking water has been reported in rats with trinitrobenzine-sulfonic acid colitis (Al-Drees and Khalil, 2016) and in C57BL/6 mice with DSS colitis (Coburn et al., 2012; Andrade et al., 2016), but the present data demonstrate for the first time that Arg supplementation in the diet is protective in two models of colonic inflammation. Importantly, it is unlikely that the very small caloric difference between the Arg^0^, Arg^NL^, and Arg^HIGH^ diets (only 0.05% higher for Arg^NL^ vs. Arg^0^, and 1.8% higher for Arg^HIGH^ vs. Arg^NL^) can explain the clinical improvement observed with the Arg-rich regimen because (i) DSS-treated mice were given the special diets for only 5 days after the DSS treatment, (ii) there was no significant weight gain in control mice fed the Arg-rich diet compared to the Arg^0^ diet, and (iii) the Orn^HIGH^ and Pro^HIGH^ diets, which contain more calories than the Arg^HIGH^ diet, did not protect mice from DSS colitis. Finally, it has been described that the daily intake of Arg in healthy humans is estimated to be around 3–4 g/day (King et al., 2008; Coburn et al., 2016; Mirmiran et al., 2016), which corresponds to ~0.04 g of Arg/kg/day, and that this consumption is not altered in patients with active UC (Coburn et al., 2016). Based on the food intake of C57BL/6 mice (Bachmanov et al., 2002) and the murine metabolic rate (Demetrius, 2005), we calculate that the Arg^NL^ diet used in the current study corresponds to an intake of ~0.18 g of Arg/kg/day. These data indicate that the usual dietary intake of Arg in most regular mouse chow is about 4-fold higher than a typical human diet. This may explain why we observed some protection with the Arg^NL^ diet as it reflects an increase compared to human consumption. Our data also suggest that when patients have diminished dietary intake, such as with IBD exacerbations, the loss of Arg availability may be deleterious, as in our Arg^0^ diet condition. We further highlight here that Arg consumption could be substantially increased in IBD patients, since 10 g/day is usually well-tolerated without side effects (Collier et al., 2005; Shao and Hathcock, 2008).

The serum and colon amino acid profiling developed in our study first demonstrated that Arg concentration in the serum mimics Arg intake in both healthy animals or in mice with colitis. Conversely, Arg content in the colon was not affected by Arg diet in control mice, but was significantly lower in DSS-treated mice on the Arg^0^ diet compared to animals receiving diets containing Arg. These data suggest that, although Arg is considered a non-essential amino acid, a lack of Arg uptake by colitis patients may affect Arg concentration at the site of inflammation and may worsen the disease since we have reported that low colitis tissue Arg levels have been shown to be associated with elevated disease activity index (Coburn et al., 2016). We also found that the concentration of Orn in the serum and colon of DSS-treated mice was affected by the various Arg diets, which is in accordance with the fact that the enzyme arginase is induced in mice with colitis (Gobert et al., 2004) as well as in patients with CD or UC (Coburn et al., 2016). Note also that Orn can be synthesized from Arg by the activity of GATM (Turer et al., 2017); to date, there is no evidence that GATM is induced in the intestine of mice or patients suffering from IBD. However, *Gatm*^−/−^ mice exhibit exacerbated colitis and a decrease of creatine synthesis by enterocytes (Turer et al., 2017), which likely means that these animals may have reduced Orn content in the intestine. These data thus suggest that both arginase and GATM are probably involved in the synthesis of Orn in the colon in response to increasing concentration of Arg. Orn is a substrate for the synthesis of polyamines and Pro (Morris, 2004; Pegg, 2016). Although we observed that the polyamine putrescine and spermidine were increased in the colon of animals with DSS colitis, we did not find an effect of Arg diet on their concentration. However, the concentration of Pro, which supports cell migration and colonic epithelial restitution *in vitro* (Singh et al., 2012), was dependent on the increased content of Arg in the diet of control mice. In this context, we sought to determine the effect of Orn and Pro supplementation on DSS colitis. No significant protective effect of these two amino acids was observed, indicating that Arg supplementation inhibits colitis independently of the arginase/GATM/OAT metabolic pathway.

Furthermore, we have shown that the Arg transporter SLC7A2 has a deleterious effect on *C. rodentium* colitis by favoring Arg uptake by enterocytes and Arg-dependent bacterial adherence (Singh et al., 2016), whereas we found here that Arg treatment protects animals from *C. rodentium*-induced colitis. These conflicting data further suggest that the protective effect of an Arg-rich diet may occur independently of Arg metabolism by host cells.

Hence, we hypothesized that Arg may affect the composition of the intestinal microbiota, which in turn can result in an improvement in colitis. First, we analyzed whether the specific AIN-76A diet changes the composition of the microbiome since we used this diet to vary Arg supplementation. Mice on the regular 5L0D diet exhibited a classical gut microbiome, characterized by a prevalence of Bacteroidetes and Firmicutes phyla. Surprisingly, when the diet was switched for the AIN-76A Arg^NL^ diet, which contains the same amount of Arg as the 5L0D, an increased prevalence of Verrucomicrobia, and of its main representative *Akkermansia*, and a concomitant decreased dominance of Bacteroidetes were observed. Because the reduced prevalence of the species *Akkermansia municiphila* has been correlated with the development of various diseases (Gobert et al., 2016; Derrien et al., 2017), understanding how the AIN-76A diet supports the emergence of this bacteria and the effect of this regimen on these pathophysiological conditions deserves further investigation.

Then, we assessed the effect of Arg in mice on the AIN-76A diet. Overall, the diversity, but not the richness, of the gut microbiota was increased in animals on the Arg^HIGH^ diet, and the lowest diversity was observed in mice on the Arg^0^ diet. From these data, we propose that the restoration of microbial diversity in the intestine using Arg supplementation contributes to the protection against colitis. Supporting this concept, a loss of diversity has been reported in the fecal and mucosal microbiome of IBD patients (Frank et al., 2007; Willing et al., 2010). The increase in the Bacteroidetes abundance with Arg supplementation is particularly interesting as (i) the prevalence of bacteria belonging to this phylum is low in patients with active CD (Seksik et al., 2003; Wang et al., 2018) and UC (Frank et al., 2007; Lepage et al., 2011; Walker et al., 2011), as well as during DSS colitis (Hudcovic et al., 2009; Nagalingam et al., 2011), and (ii) non-toxigenic *Bacteroides fragilis* protects mice from experimental colitis by inhibiting Th17 cells through the release of polysaccharide A (Mazmanian et al., 2008).

Whether the dysbiosis of the gut microbiota is the primary etiology of intestinal inflammation or simply a collateral response to the pathophysiological/immunological changes that occurs in IBD patients remains unknown. However, numerous studies have highlighted that diversity of the intestinal microbiota and increased prevalence of Bacteroidetes protect from colitis. In this context, our data emphasize that Arg supplementation could be a valuable complementary medicine for IBD patients by restoring Bacteroidetes presence and overall microbial diversity. It is now of interest to determine how Arg intake affects the microbiota, either by directly modifying trophic exchanges of the gut microbiota, or indirectly through the metabolism of Arg by host cells, which in turn may affect intestinal commensals.

## Data Availability

All datasets generated for this study are included in the manuscript and/or the Supplementary Files.

## Ethics Statement

All experiments were conducted under protocol M/08/124 approved by the Vanderbilt University IACUC and Institutional Biosafety Committee, and the Research and Development Committee of the Veterans Affairs Tennessee Valley Healthcare System. Procedures were performed in accordance with institutional policies, AAALAC guidelines, the AVMA Guidelines on Euthanasia (CO_2_ asphyxiation followed by cervical dislocation), NIH regulations (Guide for the Care and Use of Laboratory Animals), and the United States Animal Welfare Act (1966).

## Author Contributions

KS, AG, and KW conceived and designed the experiments. KS, LC, DB, MmA, MA, PL, GM, HB, MW, and MP performed the experiments. KS, MS, SD, PL, CS, AG, and KW analyzed the data. AG and KW wrote the paper.

### Conflict of Interest Statement

The authors declare that the research was conducted in the absence of any commercial or financial relationships that could be construed as a potential conflict of interest.

## References

[B1] Al-Drees A. Khalil M. S. (2016). Histological and immunohistochemical effects of L-arginine and silymarin on TNBS-induced inflammatory bowel disease in rats. Histol. Histopathol. 31, 1259–1270. 10.14670/HH-11-757 26979994

[B2] Andrade M. E. Santos R. D. Soares A. D. Costa K. A. Fernandes S. O. de Souza C. M.. (2016). Pretreatment and treatment with L-arginine attenuate weight loss and bacterial translocation in dextran sulfate sodium colitis. JPEN J. Parenter. Enteral Nutr. 40, 1131–1139. 10.1177/0148607115581374 25855577

[B3] Bachmanov A. A. Reed D. R. Beauchamp G. K. Tordoff M. G. (2002). Food intake, water intake, and drinking spout side preference of 28 mouse strains. Behav. Genet. 32, 435–443. 10.1023/A:1020884312053 12467341 PMC1397713

[B4] Barthold S. W. Coleman G. L. Bhatt P. N. Osbaldiston G. W. Jonas A. M. (1976). The etiology of transmissible murine colonic hyperplasia. Lab. Anim. Sci. 26(6 Pt 1), 889–894. 1018473

[B5] Bernstein C. N. Blanchard J. F. Kliewer E. Wajda A. (2001). Cancer risk in patients with inflammatory bowel disease: a population-based study. Cancer 91, 854–862. 10.1002/1097-0142(20010215)91:4<854::AID-CNCR1073>3.0.CO;2-Z 11241255

[B6] Coburn L. A. Gong X. Singh K. Asim M. Scull B. P. Allaman M. M.. (2012). L-arginine supplementation improves responses to injury and inflammation in dextran sulfate sodium colitis. PLoS ONE7:e33546. 10.1371/journal.pone.0033546 22428068 PMC3299802

[B7] Coburn L. A. Horst S. N. Allaman M. M. Brown C. T. Williams C. S. Hodges M. E.. (2016). L-arginine availability and metabolism is altered in ulcerative colitis. Inflamm. Bowel Dis.. 22, 1847–1858. 10.1097/MIB.0000000000000790 27104830 PMC4956554

[B8] Coburn L. A. Singh K. Asim M. Barry D. P. Allaman M. M. Al-Greene N. T.. (2018). Loss of solute carrier family 7 member 2 exacerbates inflammation-associated colon tumorigenesis. Oncogene 38, 1067–1079. 10.1038/s41388-018-0492-9 30202097 PMC6377304

[B9] Cole J. R. Wang Q. Cardenas E. Fish J. Chai B. Farris R. J.. (2009). The Ribosomal Database Project: improved alignments and new tools for rRNA analysis. Nucleic Acids Res. 37, D141–D145. 10.1093/nar/gkn879 19004872 PMC2686447

[B10] Collier S. R. Casey D. P. Kanaley J. A. (2005). Growth hormone responses to varying doses of oral arginine. Growth Horm. IGF Res. 15, 136–139. 10.1016/j.ghir.2004.12.004 15809017

[B11] Demetrius L. (2005). Of mice and men. When it comes to studying ageing and the means to slow it down, mice are not just small humans. EMBO Rep. 6, S39–S44. 10.1038/sj.embor.7400422 15995660 PMC1369270

[B12] Derrien M. Belzer C. de Vos W. M. (2017). *Akkermansia muciniphila* and its role in regulating host functions. Microb. Pathog. 106, 171–181. 10.1016/j.micpath.2016.02.005 26875998

[B13] Derrien M. Vaughan E. E. Plugge C. M. de Vos W. M. (2004). *Akkermansia muciniphila* gen. nov., sp. nov., a human intestinal mucin-degrading bacterium. Int. J. Syst. Evol. Microbiol. 54(Pt 5), 1469–1476. 10.1099/ijs.0.02873-0 15388697

[B14] Dong J. Y. Qin L. Q. Zhang Z. Zhao Y. Wang J. Arigoni F.. (2011). Effect of oral L-arginine supplementation on blood pressure: a meta-analysis of randomized, double-blind, placebo-controlled trials. Am. Heart J. 162, 959–965. 10.1016/j.ahj.2011.09.012 22137067

[B15] Engel T. Ungar B. Yung D. E. Ben-Horin S. Eliakim R. Kopylov U. (2018). Vedolizumab in IBD - Lessons from real-world experience; A systematic review and pooled analysis. J. Crohns. Colitis 12, 245–257. 10.1093/ecco-jcc/jjx143 29077833

[B16] Frank D. N. St Amand A. L. Feldman R. A. Boedeker E. C. Harpaz N. Pace N. R. (2007). Molecular-phylogenetic characterization of microbial community imbalances in human inflammatory bowel diseases. Proc. Natl. Acad. Sci. U.S.A. 104, 13780–13785. 10.1073/pnas.0706625104 17699621 PMC1959459

[B17] Gobert A. P. Al-Greene N. T. Singh K. Coburn L. A. Sierra J. C. Verriere T. G. (2018). Distinct immunomodulatory effects of spermine oxidase in colitis induced by epithelial injury or infection. Front. Immunol. 9:1242 10.3389/fimmu.2018.01242 29922289 PMC5996034

[B18] Gobert A. P. Cheng Y. Akhtar M. Mersey B. D. Blumberg D. R. Cross R. K.. (2004). Protective role of arginase in a mouse model of colitis. J. Immunol. 173, 2109–2117. 10.173/3/2109 15265947

[B19] Gobert A. P. Sagrestani G. Delmas E. Wilson K. T. Verriere T. G. Dapoigny M.. (2016). The human intestinal microbiota of constipated-predominant irritable bowel syndrome patients exhibits anti-inflammatory properties. Sci. Rep. 6:39399. 10.1038/srep39399 27982124 PMC5159846

[B20] Hardbower D. M. Asim M. Luis P. B. Singh K. Barry D. P. Yang C.. (2017). Ornithine decarboxylase regulates M1 macrophage activation and mucosal inflammation via histone modifications. Proc. Natl. Acad. Sci. U.S.A. 114, E751–E760. 10.1073/pnas.1614958114 28096401 PMC5293075

[B21] Hokari R. Kato S. Matsuzaki K. Kuroki M. Iwai A. Kawaguchi A.. (2001). Reduced sensitivity of inducible nitric oxide synthase-deficient mice to chronic colitis. Free Radic. Biol. Med. 31, 153–163. 10.1016/S0891-5849(01)00565-2 11440827

[B22] Hu F. B. Stampfer M. J. Manson J. E. Rimm E. B. Colditz G. A. Rosner B. A.. (1998). Frequent nut consumption and risk of coronary heart disease in women: prospective cohort study. BMJ 317:1341. 10.1136/bmj.317.7169.1341 9812929 PMC28714

[B23] Hudcovic T. Kozakova H. Kolinska J. Stepankova R. Hrncir T. Tlaskalova-Hogenova H. (2009). Monocolonization with Bacteroides ovatus protects immunodeficient SCID mice from mortality in chronic intestinal inflammation caused by long-lasting dextran sodium sulfate treatment. Physiol. Res. 58, 101–110. 18198984 10.33549/physiolres.931340

[B24] Johnston D. G. W. Williams M. A. Thaiss C. A. Cabrera-Rubio R. Raverdeau M. McEntee C.. (2018). Loss of microRNA-21 influences the gut microbiota, causing reduced susceptibility in a murine model of colitis. J. Crohns. Colitis 12, 835–848. 10.1093/ecco-jcc/jjy038 29608690

[B25] Kaser A. Zeissig S. Blumberg R. S. (2010). Inflammatory bowel disease. Annu. Rev. Immunol. 28, 573–621. 10.1146/annurev-immunol-030409-101225 20192811 PMC4620040

[B26] King D. E. Mainous A. G. III. Geesey M. E. (2008). Variation in L-arginine intake follow demographics and lifestyle factors that may impact cardiovascular disease risk. Nutr. Res. 28, 21–24. 10.1016/j.nutres.2007.11.003 19083383 PMC2245877

[B27] Kozich J. J. Westcott S. L. Baxter N. T. Highlander S. K. Schloss P. D. (2013). Development of a dual-index sequencing strategy and curation pipeline for analyzing amplicon sequence data on the MiSeq Illumina sequencing platform. Appl. Environ. Microbiol. 79, 5112–5120. 10.1128/AEM.01043-13 23793624 PMC3753973

[B28] Krieglstein C. F. Cerwinka W. H. Laroux F. S. Salter J. W. Russell J. M. Schuermann G.. (2001). Regulation of murine intestinal inflammation by reactive metabolites of oxygen and nitrogen: divergent roles of superoxide and nitric oxide. J. Exp. Med. 194, 1207–1218. 10.1084/jem.194.9.1207 11696587 PMC2195977

[B29] Langhorst J. Wulfert H. Lauche R. Klose P. Cramer H. Dobos G. J.. (2015). Systematic review of complementary and alternative medicine treatments in inflammatory bowel diseases. J. Crohns. Colitis 9, 86–106. 10.1093/ecco-jcc/jju007 25518050

[B30] Lepage P. Hasler R. Spehlmann M. E. Rehman A. Zvirbliene A. Begun A.. (2011). Twin study indicates loss of interaction between microbiota and mucosa of patients with ulcerative colitis. Gastroenterology 141, 227–236. 10.1053/j.gastro.2011.04.011 21621540

[B31] Liu T. C. Stappenbeck T. S. (2016). Genetics and pathogenesis of inflammatory bowel disease. Annu. Rev. Pathol. 11, 127–148. 10.1146/annurev-pathol-012615-044152 26907531 PMC4961083

[B32] Lucotti P. Setola E. Monti L. D. Galluccio E. Costa S. Sandoli E. P.. (2006). Beneficial effects of a long-term oral L-arginine treatment added to a hypocaloric diet and exercise training program in obese, insulin-resistant type 2 diabetic patients. Am. J. Physiol. Endocrinol. Metab. 291, E906–E912. 10.1152/ajpendo.00002.2006 16772327

[B33] Mannon P. J. Fuss I. J. Mayer L. Elson C. O. Sandborn W. J. Present D.. (2004). Anti-interleukin-12 antibody for active Crohn's disease. N. Engl. J. Med. 351, 2069–2079. 10.1056/NEJMoa033402 15537905

[B34] Mazmanian S. K. Round J. L. Kasper D. L. (2008). A microbial symbiosis factor prevents intestinal inflammatory disease. Nature 453, 620–625. 10.1038/nature07008 18509436

[B35] Mirmiran P. Bahadoran Z. Ghasemi A. Azizi F. (2016). The association of dietary L-arginine intake and serum nitric oxide metabolites in adults: a population-based study. Nutrients 8:E311. 10.3390/nu8050311 27213443 PMC4882723

[B36] Morris S. M. Jr. (2004). Enzymes of arginine metabolism. J. Nutr. 134, 2743S−2747S. 10.1093/jn/134.10.2743S 15465778

[B37] Nagalingam N. A. Kao J. Y. Young V. B. (2011). Microbial ecology of the murine gut associated with the development of dextran sodium sulfate-induced colitis. Inflamm. Bowel Dis. 17, 917–926. 10.1002/ibd.21462 21391286 PMC3058753

[B38] Pegg A. E. (2016). Functions of polyamines in mammals. J. Biol. Chem. 291, 14904–14912. 10.1074/jbc.R116.731661 27268251 PMC4946908

[B39] Pruesse E. Quast C. Knittel K. Fuchs B. M. Ludwig W. Peplies J.. (2007). SILVA: a comprehensive online resource for quality checked and aligned ribosomal RNA sequence data compatible with ARB. Nucleic Acids Res. 35, 7188–7196. 10.1093/nar/gkm864 17947321 PMC2175337

[B40] Rachmilewitz D. Stamler J. S. Bachwich D. Karmeli F. Ackerman Z. Podolsky D. K. (1995). Enhanced colonic nitric oxide generation and nitric oxide synthase activity in ulcerative colitis and Crohn's disease. Gut 36, 718–723. 7541008 10.1136/gut.36.5.718 PMC1382676

[B41] Rognes T. Flouri T. Nichols B. Quince C. Mahe F. (2016). VSEARCH: a versatile open source tool for metagenomics. PeerJ. 4:e2584. 10.7717/peerj.2584 27781170 PMC5075697

[B42] Sandborn W. J. van Assche G. Reinisch W. Colombel J. F. D'Haens G. Wolf D. C.. (2012). Adalimumab induces and maintains clinical remission in patients with moderate-to-severe ulcerative colitis. Gastroenterology 142, 257–265 e251–253. 10.1053/j.gastro.2011.10.032 22062358

[B43] Seksik P. Rigottier-Gois L. Gramet G. Sutren M. Pochart P. Marteau P.. (2003). Alterations of the dominant faecal bacterial groups in patients with Crohn's disease of the colon. Gut 52, 237–242. 10.1136/gut.52.2.237 12524406 PMC1774977

[B44] Shao A. Hathcock J. N. (2008). Risk assessment for the amino acids taurine, L-glutamine and L-arginine. Regul. Toxicol. Pharmacol. 50, 376–399. 10.1016/j.yrtph.2008.01.004 18325648

[B45] Shilts M. H. Rosas-Salazar C. Tovchigrechko A. Larkin E. K. Torralba M. Akopov A. (2016). Minimally invasive sampling method identifies differences in taxonomic richness of rasal microbiomes in young infants associated with mode of delivery. Microb. Ecol. 71, 233–242. 10.1007/s00248-015-0663-y 26370110 PMC4688197

[B46] Shivashankar R. Tremaine W. J. Harmsen W. S. Loftus E. V. Jr. (2017). Incidence and prevalence of Crohn's disease and ulcerative colitis in Olmsted County, Minnesota from 1970 through 2010. Clin. Gastroenterol. Hepatol. 15, 857–863. 10.1016/j.cgh.2016.10.039 27856364 PMC5429988

[B47] Singh K. Al-Greene N. T. Verriere T. G. Coburn L. A. Asim M. Barry D. P.. (2016). The L-arginine transporter solute carrier family 7 member 2 mediates the immunopathogenesis of attaching and effacing bacteria. PLoS Pathog. 12:e1005984. 10.1371/journal.ppat.1005984 27783672 PMC5081186

[B48] Singh K. Chaturvedi R. Barry D. P. Coburn L. A. Asim M. Lewis N. D.. (2011). The apolipoprotein E-mimetic peptide COG112 inhibits NF-kappaB signaling, proinflammatory cytokine expression, and disease activity in murine models of colitis. J. Biol. Chem. 286, 3839–3850. 10.1074/jbc.M110.176719 21115487 PMC3030385

[B49] Singh K. Coburn L. A. Asim M. Barry D. P. Allaman M. M. Shi C.. (2018). Ornithine decarboxylase in macrophages exacerbates colitis and promotes colitis-associated colon carcinogenesis by impairing M1 immune responses. Cancer Res. 78, 4303–4315. 10.1158/0008-5472.CAN-18-0116 29853605 PMC6072585

[B50] Singh K. Coburn L. A. Barry D. P. Asim M. Scull B. P. Allaman M. M.. (2013). Deletion of cationic amino acid transporter 2 exacerbates dextran sulfate sodium colitis and leads to an IL-17-predominant T cell response. Am. J. Physiol. Gastrointest. Liver Physiol. 305, G225–240. 10.1152/ajpgi.00091.2013 23703655 PMC3742860

[B51] Singh K. Coburn L. A. Barry D. P. Boucher J. L. Chaturvedi R. Wilson K. T. (2012). L-arginine uptake by cationic amino acid transporter 2 is essential for colonic epithelial cell restitution. Am. J. Physiol. Gastrointest. Liver Physiol. 302, G1061–1073. 10.1152/ajpgi.00544.2011 22361732 PMC3362080

[B52] Singh P. Ananthakrishnan A. Ahuja V. (2017). Pivot to Asia: inflammatory bowel disease burden. Intest Res 15, 138–141. 10.5217/ir.2017.15.1.138 28239326 PMC5323305

[B53] Terzic J. Grivennikov S. Karin E. Karin M. (2010). Inflammation and colon cancer. Gastroenterology 138, 2101–2114 e2105. 10.1053/j.gastro.2010.01.058 20420949

[B54] Torres J. Mehandru S. Colombel J. F. Peyrin-Biroulet L. (2017). Crohn's disease. Lancet 389, 1741–1755. 10.1016/S0140-6736(16)31711-1 27914655

[B55] Turer E. McAlpine W. Wang K. W. Lu T. Li X. Tang M.. (2017). Creatine maintains intestinal homeostasis and protects against colitis. Proc. Natl. Acad. Sci. U.S.A. 114, E1273–E1281. 10.1073/pnas.1621400114 28137860 PMC5321020

[B56] Ungaro R. Mehandru S. Allen P. B. Peyrin-Biroulet L. Colombel J. F. (2017). Ulcerative colitis. Lancet 389, 1756–1770. 10.1016/S0140-6736(16)32126-2 27914657 PMC6487890

[B57] Visek W. J. (1986). Arginine needs, physiological state and usual diets. A reevaluation. J Nutr 116, 36–46. 10.1093/jn/116.1.36 3080558

[B58] Walker A. W. Sanderson J. D. Churcher C. Parkes G. C. Hudspith B. N. Rayment N.. (2011). High-throughput clone library analysis of the mucosa-associated microbiota reveals dysbiosis and differences between inflamed and non-inflamed regions of the intestine in inflammatory bowel disease. BMC Microbiol. 11:7. 10.1186/1471-2180-11-7 21219646 PMC3032643

[B59] Wang Y. Gao X. Ghozlane A. Hu H. Li X. Xiao Y. (2018). Characteristics of fecal microbiota in pediatric Crohn's disease and their dynamic changes during infliximab therapy. J. Crohns. Colitis 12, 337–346. 10.1093/ecco-jcc/jjx153 29194468

[B60] Willing B. P. Dicksved J. Halfvarson J. Andersson A. F. Lucio M. Zheng Z.. (2010). A pyrosequencing study in twins shows that gastrointestinal microbial profiles vary with inflammatory bowel disease phenotypes. Gastroenterology 139, 1844–1854. 10.1053/j.gastro.2010.08.049 20816835

[B61] Yoshida Y. Iwai A. Itoh K. Tanaka M. Kato S. Hokari R.. (2000). Role of inducible nitric oxide synthase in dextran sulphate sodium-induced colitis. Aliment. Pharmacol. Ther. 14(Suppl. 1), 26–32. 10.1046/j.1365-2036.2000.014s1026.x 10807400

