# Supplementary material for: Dietary Arginine Regulates Severity of Experimental Colitis and Affects the Colonic Microbiome
Source: Front Cell Infect Microbiol. 2019 Mar 26;9:66. doi: 10.3389/fcimb.2019.00066 (PMC6443829; doi:10.3389/fcimb.2019.00066)
Supplement: Supplementary file 1 [file Data_Sheet_1.PDF]

Supplementary Material

Dietary Arginine Regulates Severity of Experimental Colitis and Affects the Colonic Microbiome

Kshipra Singh, Alain P. Gobert, Lori A. Coburn, Daniel P. Barry, Margaret Allaman, Paula B. Luis, Claus Schneider, Ginger L. Milne, Helen H. Boone, Meghan H. Shilts, M. Kay Washington, Suman R. Das, M. Blanca Piazuelo, and Keith T. Wilson*

*** Correspondence:** keith.wilson@vanderbilt.edu

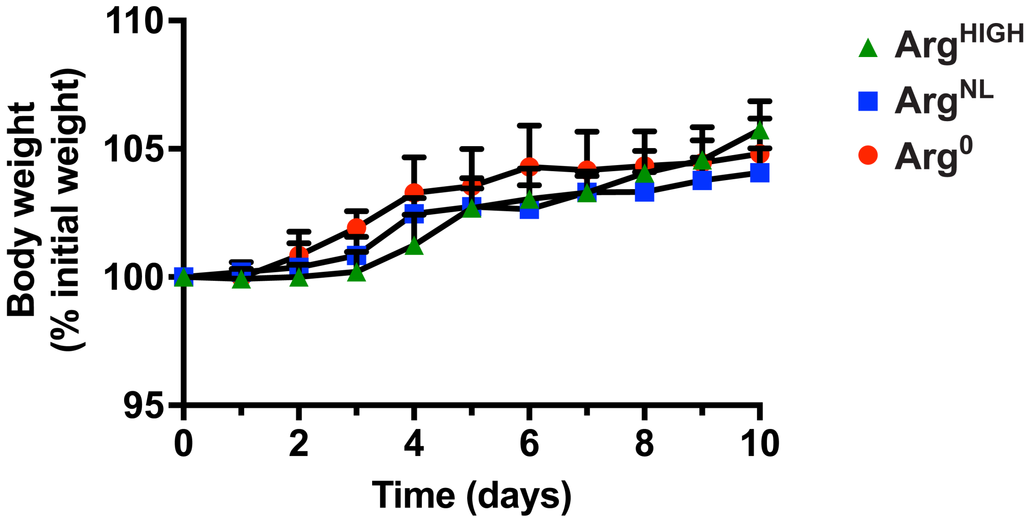

Figure S1. Effect of Arg diet on body weight. C57BL/6 mice were treated with Arg^{0}, Arg^{NL}, or Arg^{HIGH} diets for 10 days. Body weights were monitored daily and are presented as percentage of initial body weight.
