# Supplementary material for: Dietary Arginine Regulates Severity of Experimental Colitis and Affects the Colonic Microbiome
Source: Front Cell Infect Microbiol. 2019 Mar 26;9:66. doi: 10.3389/fcimb.2019.00066 (PMC6443829; doi:10.3389/fcimb.2019.00066)
Supplement: Supplementary file 2 [file Data_Sheet_2.PDF]

Supplementary Material

Dietary Arginine Regulates Severity of Experimental Colitis and Affects the Colonic Microbiome

Kshipra Singh, Alain P. Gobert, Lori A. Coburn, Daniel P. Barry, Margaret Allaman, Paula B. Luis, Claus Schneider, Ginger L. Milne, Helen H. Boone, Meghan H. Shilts, M. Kay Washington, Suman R. Das, M. Blanca Piazuelo, and Keith T. Wilson*

*** Correspondence:** keith.wilson@vanderbilt.edu

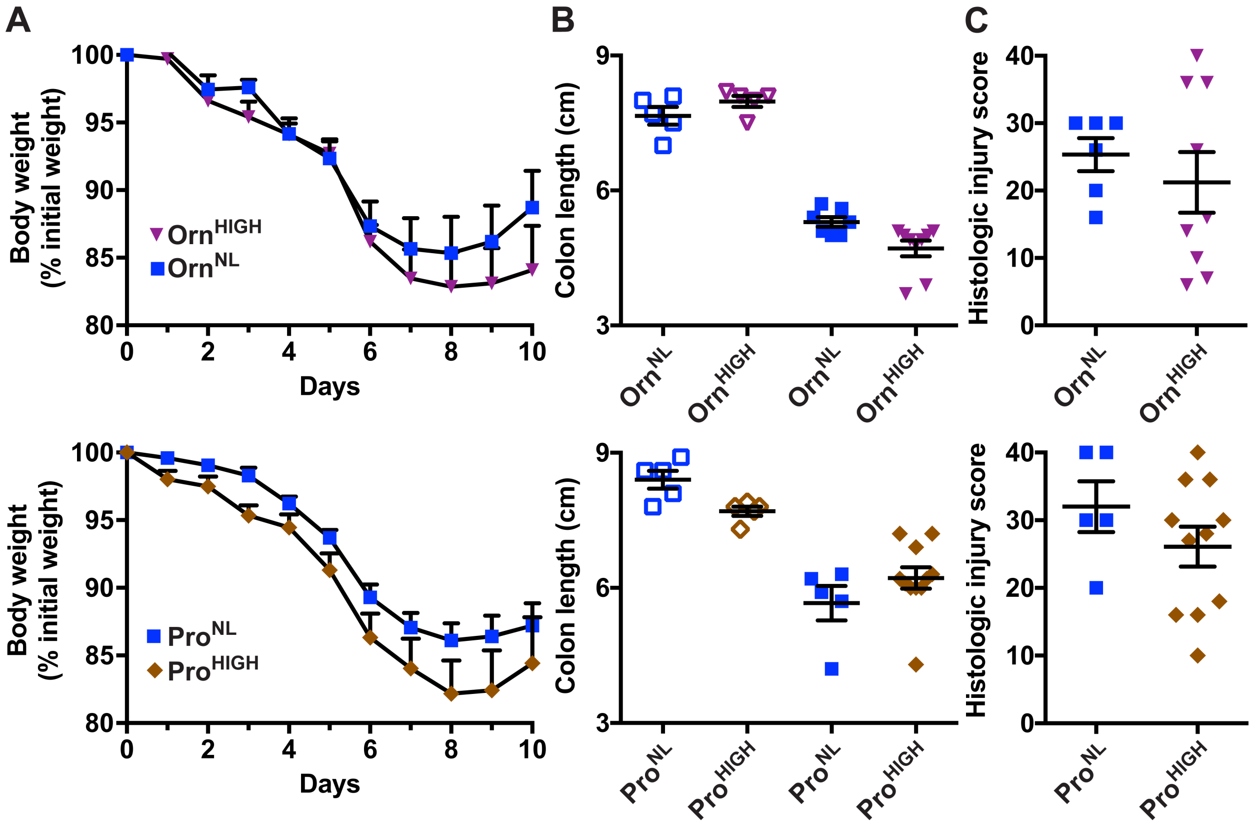

Figure S2. Effect of Orn and Pro supplementation on DSS colitis. C57BL/6 mice were treated with 2.5% DSS for 5 days and then kept for 5 more days on Orn^{NL}, Orn^{HIGH}, Pro^{NL}, or Pro^{HIGH} diets. (A) Body weights were monitored daily and depicted as percentage of initial body weight. (B) Colons were harvested and measured. (C) The histologic injury score of the colon was determined.
